# Harnessing Memory NK Cell to Protect Against COVID-19

**DOI:** 10.3389/fphar.2020.01309

**Published:** 2020-08-20

**Authors:** Saeede Soleimanian, Ramin Yaghobi

**Affiliations:** Shiraz Transplant Research Center, Shiraz University of Medical Sciences, Shiraz, Iran

**Keywords:** SARS-CoV-2, immune system, adaptive NK cell, memory NK cell, vaccination, inflammatory cytokine

## Abstract

The worldwide struggle against the coronavirus disease 2019 (COVID-19) as a public health crisis continues to sweep across the globe. Up to now, effective antiviral treatment against COVID-19 is not available. Therefore, throughout virus infections, a thorough clarification of the virus-host immune system interactions will be most probably helpful to encounter these challenges. Emerging evidence suggests that just like SARS and MERS, COVID-19 primarily suppresses the innate immune system, enabling its stable propagation during the early stage of infection. Consequently, proinflammatory cytokines and chemokines have been increasing during infection progression associated with severe lung pathology. It is imperative to consider hyper inflammation in vaccine designing, as vaccine-induced immune responses must have a protective role against infection without leading to immunopathology. Among the front-line responders to viral infections, Natural Killer (NK) cells have immense therapeutic potential, forming a bridge between innate and adaptive responses. A subset of NK cells exhibits putatively increased effector functions against viruses following pathogen-specific and immunization. Memory NK cells have higher cytotoxicity and effector activity, compared with the conventional NK cells. As a pioneering strategy, prompt accumulation and long‐term maintenance of these memory NK cells could be an efficacious viral treatment. According to the high prevalence of human cytomegalovirus (HCMV) infection in the world, it remains to be determined whether HCMV adaptive NK cells could play a protective role against this new emerging virus. In addition, the new adaptive-like KIR+NKG2C+ NK cell subset (the adaptive-like lung tissue residue [tr]NK cell) in the context of the respiratory infection at this site could specifically exhibit the expansion upon COVID-19. Another aspect of NK cells we should note, utilizing modified NK cells such as allogeneic off-the-shelf CAR-NK cells as a state-of-the-art strategy for the treatment of COVID-19. In this line, we speculate introducing NKG2C into chimeric antigen receptors in NK cells might be a potential approach in future viral immunotherapy for emerging viruses. In this contribution, we will briefly discuss the current status and future perspective of NK cells, which provide to successfully exploit NK cell-mediated antiviral activity that may offer important new tools in COVID-19 treatment.

## Introduction

World Health Organization declared the outbreak of coronavirus disease 2019 (COVID-19) a Public Health concern on 30 January 2020 (Velavan and Meyer, 2020). This novel coronavirus disease pandemic caused by the severe acute respiratory syndrome coronavirus 2 (SARS-CoV-2) that has led to a global struggle to cope with this public health crisis (Van Bavel et al., 2020). SARS-CoV-2 virus preferentially impacts the cells in the respiratory system, but the direct effects of its damage on other organs including heart, liver, brain, and kidneys have also been reported in patients with SARS-CoV-2 infection (Prasanna and Abilash, 2020). An unanswered question has been currently raised why SARS-CoV-2 can result in a wide variety of clinical manifestations ranging from: asymptomatic, mild, moderate, to severe states in COVID-19 patients, a major challenge in the management of medical decisions and interventions (Gandhi et al., 2020; Meini et al., 2020; Michelen et al., 2020). The severe cases of COVID-19, have accompanied pneumonia, which can progress to acute respiratory distress syndrome (ARDS), sepsis, septic shock, and multi-organ failure (particularly kidney, heart, and liver damage) (Cao, 2020). Obviously, the pathogenesis of SARS-CoV-2 is largely related to its interplay with the host. Indeed, the interaction between SARS-CoV-2 and host antiviral immunity, including innate and acquired immune response, should be investigated (Ahmadpoor and Rostaing, 2020; Ranucci et al., 2020). On the basis of current COVID-19 data, cytokine storm and therefore the development of ARDS are due to the exaggerated immune responses leading to severe lung damage (Zhang et al., 2020). Up to now, there are no specific and effective COVID-19 therapies. At this time, it is our opinion that immunotherapies based on immunomodulation and counterbalancing of inflammatory cytokine could reduce inflammation and inflammatory associated lung injury.

In this regard, Natural Killer (NK) cells as essential front-line responders to many viral infections in humans have been proposed for a suitable therapeutic approach in severe COVID-19 patients, and several clinical trials have begun (Market et al., 2020). In this study, we considered this new approach to enhance patient’s survival using NK cells with memory features and immunomodulatory potential and discussed also the aspects of this proposed treatment.

## Immunological Aspects of Sars-Cov2 Features, Diagnosis, And Treatment

Coronaviruses(CoVs) belong to the Coronaviridae family and are characterized by a positive-sense single-strand ribonucleic acid (RNA). The coronavirus genome is highly susceptible to mutations that result in genetic drift and evade immune recognition (Kikkert, 2020). Consistent with this notion, SARS-CoV-2, as a new member of the genus Beta coronaviruses, can escape from immune detection during the infection (Channappanavar and Perlman, 2017; Prompetchara et al., 2020) and worsening disease outcome could be associated with the immune-escape mechanisms behind these chronic diseases. Indeed, the immune homeostasis is disrupted by SARS-CoV-2 leading to declined responsiveness of host immune regulatory system, macrophage function, and alterations in lymphocyte subsets (Merad and Martin, 2020; Wan et al., 2020).

### Confronting Immune Response With SARS-CoV-2

Accumulating evidence suggests that the stimulated immune response by SARS-CoV-2 infection results in two phases; first, immune defense-based protective phase at the early stage, and second, severe-stage inflammation-driven damaging phase (Alberici et al., 2020). Once the SARS-CoV2 enters the body, the innate immune system performs as a first responder for the detection of viral infections. Innate immune cells produce proinflammatory cytokines to inhibit viral replication and induce the adaptive immune response (Koyama et al., 2008). Among the initial immune responses in the fight against respiratory RNA viruses, such as Coronavirus, detection of evolutionarily conserved microbial structures, known as pathogen-associated molecular patterns (PAMPs) are ensured through germline-encoded pattern recognition receptors (PRRs) (Bowie and Unterholzner, 2008). PRRs including: TLR3, TLR7, TLR8, MDA-5, and RIGI receptors are produced by tissue resident macrophages and dendritic cells (DCs) (Kumar and Carmichael, 1998; Kawai and Akira, 2006). This results in the activation of transcription factors involved in the production of type I interferons (IFNs) (IFN-α/β). In this line, Type I IFNs have a critical role in concert with pattern PRR signaling to prime innate and adaptive antiviral responses such as stimulating natural killer (NK) cells, macrophages, and production of proinflammatory cytokines (Samuel, 2001; Murira and Lamarre, 2016).

### Immunopathogenesis of SARS-CoV-2

**Figure 1:**
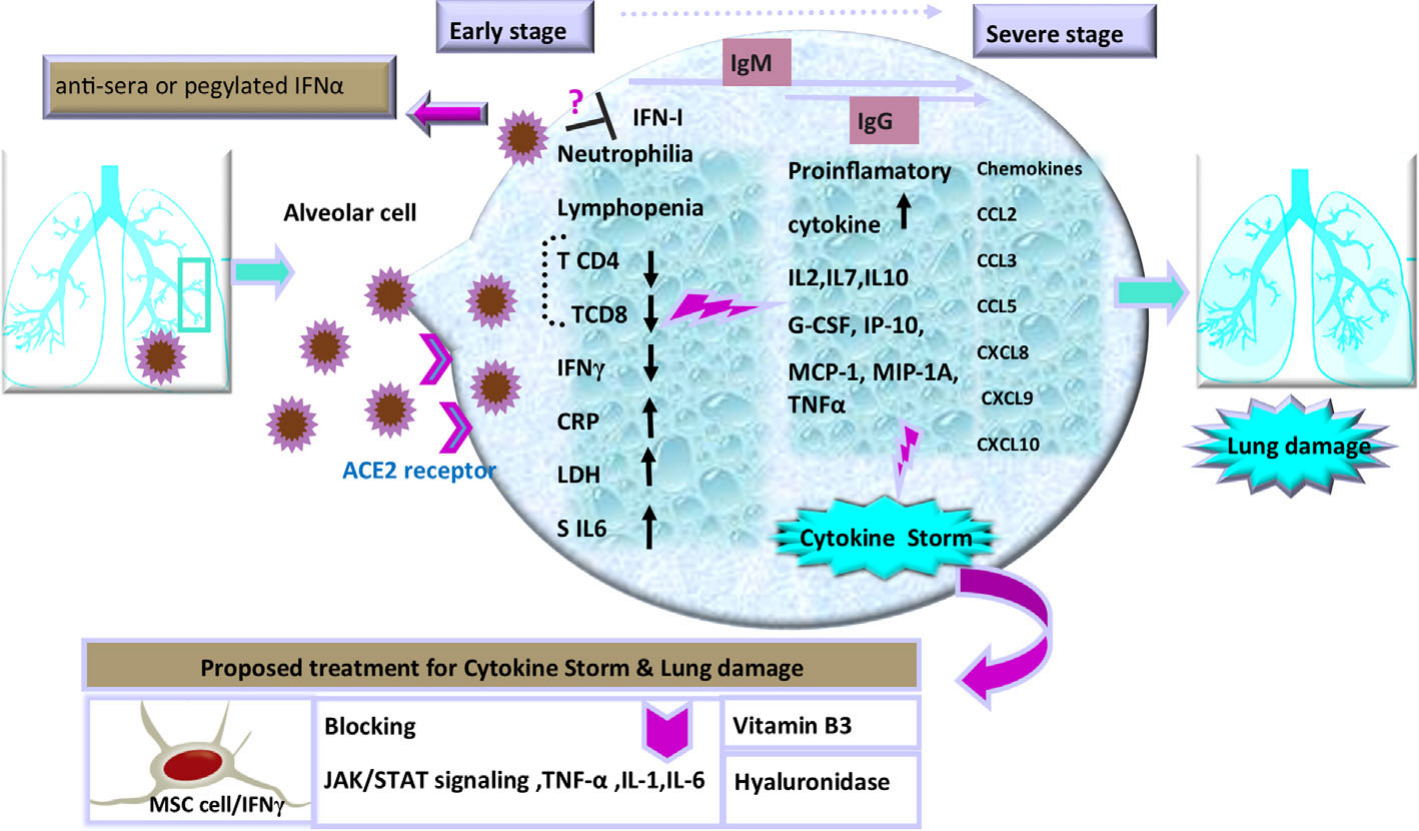
Schematic representation of the progression of COVID-19 and potential interventions. SARS-CoV-2 enters cells through interactions with receptors that include angiotensin converting enzyme II (ACE2), a cell surface protein on the cells. The increased concentrations of the serum cytokines and chemokines is correlated with the disease severity and adverse clinical outcome. There are some proposed therapeutic options in the management of cytokine storm and pulmonary injury, including: use of cytokine activated mesenchymal stem cells, Vitamin B3, and Hyaluronidase. Inhibition of TNF-α, IL-1, or IL-6 to manage COVID-19-induced cytokine release syndrome (CRS) is also recommended.

The immunomodulatory strategies that are used by beta coronaviruses can lead to dysregulated IFN responses (Blanco-Melo et al., 2020). Similar to SARS and MERS, SARS-CoV-2 replicates silently in host cells with suppressed IFNs response, leading to high viral loads (Acharya et al., 2020; Rokni et al., 2020). By contrast, following limited production of IFNs, the robust production of pro-inflammatory cytokines and chemokines may occur after an enhanced recruitment of neutrophils to the lungs (Channappanavar et al., 2019). The virus actively subverts IFNs to manipulate the host cytokine environment for its benefit. During the early phase, a specific immune response is required to clear the virus and to impede disease progression to severe stages. When a protective immune response is impaired, the virus will propagate and cause massive damage to the organs expressing the surface receptors angiotensin-converting enzyme 2 (ACE2). These are recognized by alveolar macrophages and epithelial cells, triggering the generation of pro-inflammatory cytokines and chemokines including: IL-2, IL-7, IL-10; granulocyte colony-stimulating factor (G-CSF), interferon gamma-induced protein 10 (IP-10), and monocyte chemoattractant protein-1 (MCP-1) in the damaged cells (Xu et al., 2020). Moreover, such evidence has demonstrated elevated levels of macrophage inflammatory protein 1 alpha (MIP-1A), tumor necrosis factor-alpha (TNF-α), as well as chemokines (C-C motif chemokine ligand 2: CCL2, CCL3, CCL5, C-X-C motif chemokine ligand 8: CXCL8, CXCL9, CXCL10) (**Figure 1**) in the severe stage of this viral disease (Di Lernia, 2020). Increasing concentration of these proteins results in the production of IFNγ by T cells and monocytes during virus infection (Mardani et al., 2020; Tay et al., 2020; Yuan et al., 2020). Hence, excessive cytokine/chemokine release can cause hyperinflammation, which leads to severe complications of the disease including pneumonitis, acute lung injury (ALI), ARDS, shock, vascular hyper permeability, organ failure, and death (Henderson et al., 2020; Mehta et al., 2020).

Thus, it is essential to diagnose the infection during the first phase before the cytokine storm is initiated (Nicholls et al., 2003; Mehta et al., 2020). In addition, the protective defense during the early stages of viral infections is of utmost importance to rein the spread of the viruses and it is imperative to boost immune responses (anti-sera or pegylated IFN-α) at this stage. In this regard, an experimental trial has established that recombinant human IFN-α (rhIFN-α) nasal drops (**Figure 1**) in susceptible healthy people are able to potentiate protection against COVID-19 pandemic (Shen and Yang, 2020). Thus, the timing of treatment administration in infection control is a key to yield protective responses (Channappanavar and Perlman, 2017; Chen J. et al., 2020; Wu et al., 2020).

### Immunological Diagnostic Tools for SARS-CoV2 

Evaluation of diagnostic accuracy for COVID-19 is still in progress, so an authentic interpretation of the findings in this area is essential in guiding patient care. Indeed, clear pattern of inflammatory and immune biomarker abnormalities is needed to discriminate the stages of infection, which may potentially aid in addition to molecular detection of virus. The detection of both SARS-CoV-2 nucleic acid and specific antibodies to viral proteins have thus far become significant for primary diagnosis infection and immunity in COVID-19 patients, respectively. Two types of reliable diagnostic tests have been thus far commonly utilized including reverse transcriptase-polymerase chain reaction (RT-PCR) and immunoglobulin M (IgM) and IgG antibody testing (Nalla et al., 2020; To et al., 2020). The RT-PCR has been the key diagnostic test using upper respiratory tract specimens or nasopharyngeal swabs. At an early stage, before the launch of adaptive response, IgM has been further detected in patient blood after 3–6 days and then IgG responses that are important as a long-term immunity and immunological memory, reported after 8 days (Li Z. et al., 2020). Notably, both asymptomatic and symptomatic patients have shown variations in the course of PCR positivity and seroconversion (Sethuraman et al., 2020). Accordingly, identification of hematologic, biochemical, and especially immunologic biomarkers can be helpful in guiding patient care decisions. Assessment of biomarkers including neutrophil and lymphocyte counts, neutrophil-to-lymphocyte ratio (NLR), CRP, troponin T (TnT), D-dimer, IL-6, IL-2R,IL-8,IL-10 (Henry et al., 2020), ferritin, lactic acid dehydrogenase (LDH), and procalcitonin (PCt) as a predictive of disease progression beside clinical manifestations and molecular diagnosis can be beneficial for making accurate decisions to fulfill antiviral and anti-inflammatory interventions (Fang et al., 2020; Henry et al., 2020).

### Immunotherapeutic Approach of SARS-CoV2

A few weeks after COVID-19 outbreak, several science groups started to design a vaccine for this novel disease. SARS-CoV and MERS-CoV immunization studies have also become a pattern for targeting antigen selection in designing vaccines (Du et al., 2009; Al-Amri et al., 2017). Moving forward, knowledge of developing effective therapeutics for COVID-19 is necessary to make a balance between inflammatory cytokine secretion and antiviral properties in immune cell therapy. Therefore, controlling deleterious rather than protective role of lymphocyte responses should be taken into account. In this regard, recent evidence has suggested some effective strategies including blocking IL-6 (tocilizumab) (Tanaka et al., 2016; Monteleone et al., 2020), IL-1 (anakinra), and TNFs to calm inflammatory storm (Fu et al., 2020; Shi et al., 2020). Selective cytokine blockade could be a potential treatment to reduce the mortality of severe COVID-19 (Shakoory et al., 2016; Coomes and Haghbayan, 2020; Mehta et al., 2020). Moreover, another therapeutic option has been Janus kinase (JAK) inhibitor, tofacitinib, denoting that blocking JAK/STAT signaling can reduce cytokine storm as an anti-IL-6 operator (Henderson et al., 2020; Liu et al., 2020). It should be noted that despite the benefits of these anti-inflammatory agents, there are some disadvantages, such as tendency for general infections and the development of chronic inflammatory disorders, thus more experimental investigations should be done to clarify doubts (Jones and Ding, 2010; McGonagle et al., 2020; Soy et al., 2020). Furthermore, in response to inflammatory cytokines, lungs are filled with a clear liquid jelly. Actually, the nature of the clear jelly is hyaluronan (HA). Thus, inhibition of hyaluronan synthase (HAS) and elimination of this jelly by hyaluronidase has been recommended (Modig and Hällgren, 1989). In addition, numerous studies have suggested that vitamin B3 (i.e., niacin or nicotinamide) can be highly effective in preventing lung tissue injury in animal models with bleomycin-induced lung injury (**Figure 1**), which might be another approach to supply this food supplement to COVID-19 patients (Nagai et al., 1994; Shi et al., 2020). These treatment approaches play auxiliary roles in severe patients of COVID-19. Likewise, on the basis of new findings, the potential benefits or disadvantages of anti-cytokine therapy has implications for other therapeutic strategies such as Cellular therapy in patients with severe inflammatory responses. Cellular therapy as an effective treatment for severe COVID-19 patients is a rapidly evolving field with novel constructs being developed. Various clinical sites in China have revealed that mesenchymal stem cells (MSCs) in severe COVID-19 infection can be nominated as a helpful mode in the suppression of hyperactive immune response and promotion of tissue repair. As well, MSCs with IFNγ as licensed-MSCs can be more effective (Wang et al., 2014; Wang et al., 2018). Besides, current investigations have strongly indicated that T-cell response can be a crucial strategy for the control of SARS-CoV and MERS-CoV and probably proper for SARS-CoV-2; nevertheless, cytokine storm should be controlled in order not to cause lung pathology. Indeed, one of the biggest obstacles in severe COVID-19 cell therapy approach (such as chimeric antigen receptor: CAR T-cell) is the cytokine release syndrome (CRS) affecting patients with severe conditions (Maude et al., 2014). As well, IFN-γ production is indispensable for resistance against virus infection, but T-cells as a major source of IFN-γ in the adaptive immune response take days to develop a prominent IFN-γ response (Chen G. et al., 2020). Remarkably, it is important to consider it in vaccine designing, as vaccine-induced immune responses need to play a protective role against infection without leading to immunopathology with timely management of treatments. It is noteworthy that the abolition of viruses requires the immune system to respond through a programmed regulatory system, subsequently to implement tolerance and to inhibit excess response. Indeed, it must be balanced between sufficient inflammatory reactions against pathogen and overactive cytokine stimulation. Among the immune cells, NK cells endowed with significant strategies for self-tolerance status while allowing efficacy against viral attacks. Consistent with this context, education is one of the immunomodulatory process during NK cell development, operating not as an on-off switch but as a rheostat, tuned by a quantitative influence on individual NK cells (Orr and Lanier, 2010; Vivier et al., 2011). Intriguingly, this pattern has implications for the use of NK cells in therapeutic settings and affects interpretations of how NK cells control virus infections and regulate autoimmunity. Additionally, NK cells act as a rheostat by removing activated CD4+ and CD8+ T cells in viral infections such as HIV and HCV, thus preventing T cell-mediated autoimmunity (Lodoen and Lanier, 2005; Sun and Lanier, 2008; Frutoso and Mortier, 2019). Based on this knowledge we will comment on immune modulating treatment options that are being a proper strategy for the current COVID-19 crisis.

## NK Cell-Mediated Antiviral Mechanisms

NK cells are at the forefront of the antiviral response (Tang et al., 2013) and they are known as a regulator of immunological procedures including viral defense and immunological homeostasis. Actually, NK cells make use of various recognition modes in viral infections. The functional outcome of NK cells is determined by integrating both activating and inhibitory signals which regulate NK cell activity (Lanier, 2005; Duev-Cohen et al., 2016). Virus-infected cells expressing defined ligands can be directly targeted through activating receptors such as NK group 2-member D (NKG2D), NK cell p46-related protein (NKp46), and NKp30 on NK cells. As well, release of cytokines such as INFs, IL-12, or IL-18 by accessory cells can also alert bystander NK cells during viral infection (Cerwenka and Lanier, 2001; French and Yokoyama, 2003; Hammer et al., 2018b) and render NK cells to proliferate and produce cytokine such as IFN-γ (Zwirner and Domaica, 2010).

Another mode of recognition includes expressing Fc receptors (i.e., CD16 or FcγRIII), by NK cells identifying infected cell bound to antibodies in the surface of infected target cells, called antibody-dependent cell-mediated cytotoxicity (ADCC), resulting in the release of cytotoxic factors such as perforin and proteases known as granzyme. Therefore, NK cells can also kill virus-producing cells through this important property (Vanderven et al., 2017). Virus-infected cells usually escape T-cell immune surveillance through down-regulating the expression of major histocompatibility complex (MHC) class I to compromise for antigen presentation pathway, making viruses difficult to be recognized by T-cells (Pircher et al., 1990; McMichael, 1998). In addition, several lines of evidence have further supported the notion that NK cells play a crucial role in the first checkpoints of encountering antigens and trigger pro-inflammatory immune responses and they provide a critical response against infectious agents during the time that the acquired immune system is still being mustered (Andoniou et al., 2006; Hu et al., 2019). These features suggest that NK cells can serve as the major antiviral effector cells wherein virus-infected cells should develop mechanisms of escaping T-cell surveillance (Zander et al., 2019). Likewise, they are at the interface between innate and adaptive immunity, adjusting the activity of other immune cells through the release of cytokines including activating (e.g., IFN-γ and TNF-α) as well as inhibitory cytokines (e.g., IL-4 and IL-10) for different purposes. For instance, NK cells promote the maturation of dendritic cell (DC) through the release of IFN-γ and TNF-α (Shariat et al., 2014; Moravej et al., 2017). Additionally, NK cells are considered the important effectors in shaping the subsequent immune responses. They increase or decrease function of immune cells through IFNγ or IL-10 secretion, respectively (Vivier et al., 2011; Della Chiesa et al., 2019; Monica et al., 2019).

**Figure 2:**
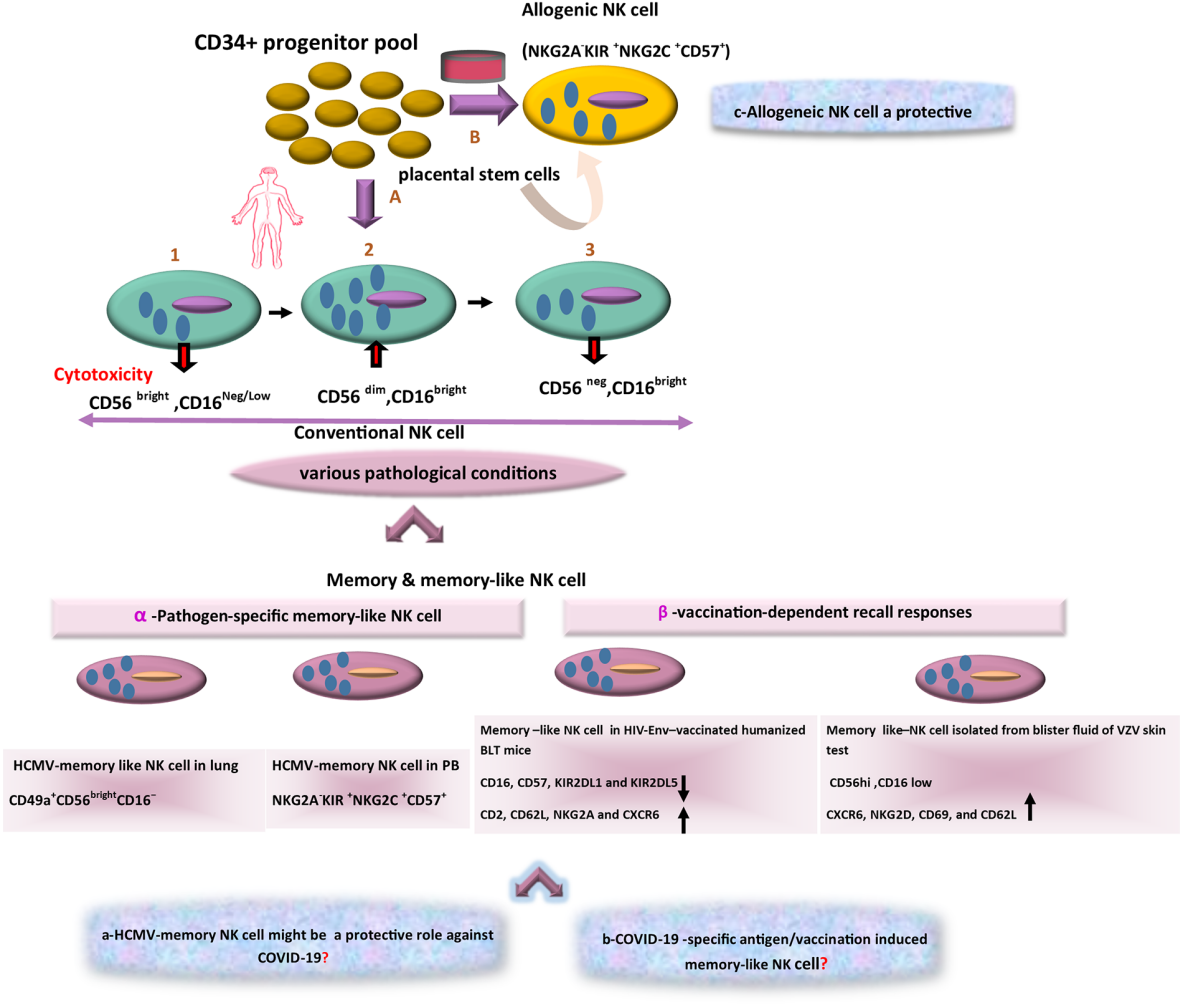
The scheme of different aspects of NK-based adoptive therapy in COVID-19. In the human body, Conventional NK cells have different subsets with phenotypic diversity. Based on various pathological conditions, distinct memory NK cells that are generated in peripheral blood (PB) and tissues. Response to specific viral antigens (e.g., in human cytomegalovirus [HCMV] infection) and vaccination-dependent recall responses (e.g., in HIV-Env–vaccinated humanized BLT mice and blister fluid of VZV skin test) lead to expansion of memory-like NK cells in PB and various tissues. The question marks indicate events that are still hypothetical: a) Might these memory NK cells (HCMV-adaptive NK cells) be a target in treatment of COVID-19 as a cross-reactive immune response? b) Is there a distinct antigen-specific-memory NK cell against COVID-19? c) Could alloreactive NK cells become an effective cell therapy approach in COVID-19?

The diversity of NK cell function and phenotype is accordingly an issue of growing attractiveness. With reference to cytolytic activity, cytokine secretion, and organ-specific localization, three main subsets of NK cells can be consequently identified. Large amounts of cytokines such as IFNγ are thus produced by the CD56bright/CD16low subset in response to activation, whereas CD56low/CD16high cells are the major cytotoxic populations. Cytotoxicity and cytokine secretion are further regulated by making a balance between inhibitors and activating receptors and the effector function of NK cells also relies on the incorporation of activating and inhibitory receptors (Björkström et al., 2010). A third NK cell subset is CD16+/CD56neg NK cells with different features because of less functionality and lower expression of natural cytotoxicity receptors (NCRs). Evidence also suggests that CD56low NK cells originate from CD56high ones (**Figure 2**). The given subset is expanded in viral infection and then counterbalances the reduction in CD56dim/CD16pos cytolytic NK cell subset (Mikulak et al., 2017). In general, these various recognition modes trigger NK cells activation, and then their cytotoxicity function that results in the lysis of pathogen-infected target cells (Hammer et al., 2018b). Although there are numerous ways to recognize viral antigens and peptides by NK cells, the implications of such cells should be considered in the control of viral infections.

### NK Cells-Mediated Antiviral Immune Response to Coronaviruses

Therapeutic strategies for COVID-19 based on the present investigations of SARS-CoV and MERS-CoV infections may be helpful in offering potential treatment targets for fighting this pandemic.

In this regard, the National Research Project for SARS, Beijing Group, has reported that in the cohort study of 221 patients with SARS who were admitted to hospitals, determining the total number of NK cells and CD158b+ (KIR2DL3) NK cells in 72 cases of severe SARS were significantly lower than those in mild cases (National Research Project for SARS, Beijing Group, 2004). More importantly, this study has shown that percentage of CD158b+ NK cells in patients with *M pneumonia* infection and SARS patients (with positive clinical criteria and negative anti-SARS coronavirus) wasn’t significantly altered as compared to the percentages of NK cells in SARS cases (with positive for both clinical criteria and anti-SARS coronavirus), which indicates that the NK cells monitoring should be helpful in differentiating true SARS from false SARS and *M pneumonia* infection. Due to the greatest similarity of COVID-19 to SARS-CoV, NK cells monitoring can be investigated for this novel coronavirus (novel-CoV) (Bai et al., 2020; Chen N. et al., 2020). In addition, one of the CoVs in pigs, i.e., transmissible gastroenteritis virus (TGEV) had been first described in 1946 in which increasing infection in young pigs could result from lack of NK cell activity against TGEV-infected cells. Studies have further suggested that IFN might be needed to activate NK cells in newborn pigs, contributing to resistance to challenges with TGEV (Cepica and Derbyshire, 1984; Lesnick and Derbyshire, 1988; Loewen and Derbyshire, 1988; Annamalai et al., 2015), and they have further revealed that NK cells might play a significant role in fighting CoVs.

Other studies, consistent with this context, have shown severe cases of SARS patients with a lower level of NK cells than those in milder cases (Cui et al., 2003; Peiris et al., 2003; Wong et al., 2003). Likewise, in the study by J Chen et al. in a pneumonia model of SARS in mice, mimicking features of the human disease, illustrated that mice depleted of both CD4 and CD8T cells, had the ability to control SARS-CoV replication in the lungs, suggesting an immune mechanism independent of T cells, and a role for innate antiviral response and NK cells, in viral clearance. It should be noted that at the early phase of infection, NK cells, macrophages, and plasmacytoid DCs (pDCs) could migrate into the lungs. This can occur following the first wave of enhanced expression of cytokines including TNF-α, IL-6, and CXCL10, CCL2, CCL3, and CCL5, as virus titers, produced by airway epithelial cells and alveolar macrophages (Chen et al., 2010). Indeed, the activation of the innate immune system and NK cells has a significant role in the primary phase of infection in the control of SARS-CoV replication. This approves the hypothesis that in critically ill patients with sepsis, the activation of NK cells is an important element to combat infections. But, continuous activation of NK cells in sepsis causes exhaustion and hyporesponsiveness of NK cells which results in hyper inflammatory response due to reduced NK cell numbers (Guo et al., 2018). On the contrary, in the context of other respiratory viral infections like respiratory syncytial virus (RSV) (Li et al., 2012), influenza A virus (Abdul-Careem et al., 2012; Zhou et al., 2013), NK cells appear to cause enhanced inflammation and immunopathology during infections. Furthermore, during influenza infection, stimulation, and migration of lymphocytes such as NK cells to the lungs, influence the IFN-γ production and hyper inflammatory response. Increased recruitment of hyper-responsive NK cells would thus far worsen the infection outcomes (Vivier et al., 2011; Scharenberg et al., 2019). Collectively, considering this duality of NK cell roles, we must be aware of the potential and challenges mainly the timing of cell therapy and the stage of infection to finely tune NK cell activity to facilitate viral clearance while impeding the harm of inflammatory responses.

### NK Cells-Mediated Antiviral Immune Response to SARS-CoV2

Remarkably, the total number of NK cells, as well as B-cells and T-cells, can decrease significantly in patients with COVID-19 particularly in severe cases, compared to non-severe ones (Cao, 2020). In this respect, several studies have identified reduced numbers of NK cells in the peripheral blood of COVID-19 patients, which is related to the severity of the disease (Masselli et al., 2020; Vabret et al., 2020; Wang et al., 2020b). In the study by Fan Wang et al. on COVID-19, the incidence rate of lymphopenia had been reported by 72% that was consistent with SARS and MERS. CD4+ T-cells, CD8+ T-cells, and B-cells had been also decreased in 29, 29, and 25% of patients, respectively. But, NK cells has been prominently reduced in 59% of the cases (higher incidence rate than others) (Wang et al., 2020b). Accumulating evidence had supported the fact that NK cells had reduced in COVID-19 patients as compared with non-infected cases (Chen G. et al., 2020; Wen et al., 2020; Wilk et al., 2020). Of note, a latter study by Feng Wang et al. (Wang et al., 2020a) has been reported that CD4+ T cells were increased in patients with COVID-19, whereas the number of NK cells was decreased in the severe stage of infection. The important finding of this study is that the pathogenesis of severe SARS-CoV-2 infection is associated with the hyper function of CD4+ and CD8+ T cells but NK cell hyporesponsiveness. As well, new findings suggest that during COVID-19 infection, production of IgG-subclasses IgG1 and IgG3 may trigger NK-cell mediated ADCC through the IgG-Fc-receptor (FcγR) IIIa, and NK cells utilize the Fc domain of bound IgG to recognize extracellular virions or surface antigens expressed on infected cells. The lysis of infected cells and cytolytic functions by NK cells might be stimulated by this interaction (Dokun et al., 2001; Acharya et al., 2020; Bonam et al., 2020). Additionally, a recent study found ADCC mediated by NK cells, can contribute to viral control in COVID-19 patients. They observed efficient SARS-CoV-2 S glycoprotein (S309- and S306), triggering ADCC in SARS-CoV-2 S-glycoprotein-transfected cells (Pinto et al., 2020).

Interestingly, NK cells have a considerable role in inducing the lysis of virus-infected cells since the cytotoxicity of such cells is regulated by activating and inhibitory receptors. An increased NK group 2 member A (NKG2A) expression as a heterodimeric inhibitory receptor can also result in functional exhaustion of NK cells in patients with SARS-CoV-2 infection. Indeed, the total number of NK cells declines during the COVID-19 and the number of NK cells is simultaneously increased with the reduced expression of NKG2A after recovery (Yaqinuddin and Kashir, 2020; Qin et al., 2020), while this is associated with significantly decreased expression of the activation markers including CD107a, IFN-γ, IL-2, and TNF-α in T and NK cells of patients with severe COVID-19 (Antonioli et al., 2020; Zheng et al., 2020). In this respect, a study by Vabret et al. revealed crosstalk between monocytes and NK cells that results in NK cell recruitment from the peripheral blood to the lungs and killing of SARS-CoV-2-infected cells. Majority of human lung NK cells are non-resident and the expansion of CXCR3 ligands (CXCL9-11)-producing monocytes causes NK cell infiltration into the lung of COVID-19 disordered patients (Chu et al., 2020; Vabret et al., 2020). However, a question remains at this time how the SARS-CoV2 virus alters the number and function of NK cells. It is possible that NK cell redistribution in infected sites (NK cell infiltration into the lung) or apoptosis of these cells results in their reduction (McKechnie and Blish, 2020). Moreover, elevated IL-6 and IL-10 levels in COVID-19 cases can suppress NK cell activity. Further, moderating the expression of the activating receptor NKG2D through IL-6 production leads to impairment of NK cell activity. (Osman et al., 2020). In addition to elevated IL-6 and IL-10, the higher levels of TGF-β is responsible for suppressing NK cell antiviral activity through down-modulation of NKG2D (Lee et al., 2004; Huang et al., 2005). Thus, therapies targeting IL-6, IL-10, and TGF-β could be investigated as means of improving NK cell antiviral immunity in cases of COVID-19. However, the utility of therapeutic tools to boost NK cell functions should, thus, be carefully considered in severely affected patients (Rajaram et al., 2020).

## NK Cells and Immune Memory in Viral Infection

Over recent years, a novel concept has emerged indicating that the present paradigm that T-cells and B-cells are the only factors of adaptive immunity should be reconsidered. Although, NK cells have been respected for their ability to mediate spontaneous cytotoxicity during innate immune responses in an antigen-independent manner, it has become recently clear that they display long-lived immunological memory responses (Lee et al., 2015). In mice, it has been initially described that cytomegalovirus infection drives adaptive features of NK Cells with altered effector function, where NK cells bearing Ly49H receptors expanded and provided stronger responses after a secondary encounter with the virus. Intriguingly, human cytomegalovirus (HCMV) infection can also prompt emergence and expansion of adaptive or memory-like NK cells with more potent cytotoxicity in humans (Lam and Lanier, 2017). Moreover, adaptive NK cells are characterized by expression of the activating receptor NKG2C and the absence of the inhibitory receptor NKG2A. Notably, non-classical MHC-I molecule, HLA-E is a ligand for both but, NKG2A has the higher affinity for this ligand engagement in competition with NKG2C. Thus, the inhibition of NKG2A expression is an essential element for expansion of NKG2C+NKcells with the more cytolytic activity (Guo et al., 2018).

This distinct population of NK cells also provides stronger protective immunity against HCMV *via* ADCC in the presence of anti-HCMV antibodies. Moreover, adaptive NK cells are characterized by expression of CD57 (maturation marker), KIRs, and the absence of the inhibitory receptor NKG2A and can further produce inflammatory cytokine and cytolysis in response to target cell recognition (Lopez-Verges et al., 2010; Lopez-Vergès et al., 2011). Intriguingly, adaptive NK cells additionally share notable similarities in epigenetic patterns with CD8+ effector T-cells and remarkable matches in adaptive cell differentiation as well (Tesi et al., 2016).

### Memory-Like NK Cells Expansion in Various Pathological Conditions

Recenly, it has been also indicated that uterine mucosa, liver, skin, adrenal gland, colorectal and visceral adipose tissues, as well as secondary lymphoid compartments (SLCs), encompass CD56bright/CD16neg/dim NK cells, but CD56dim/CD16 bright cells settle in tissues such as lung and breast (Carrega et al., 2014; Melsen et al., 2016). It should be noted that localization of NK cell subsets changes in various pathological conditions e.g. in the presence of tumors or virus-infected cells (Nikzad et al., 2019). Even with the acquisition of new chemokine receptors by NK cells in a tumor or virus-infected cell microenvironments, NK cells may be changed functionally and phenotypically (Das and Khakoo, 2015; Brillantes and Beaulieu, 2020). Most importantly, NK cells differentiate into long-lived memory ones. For instance, in humans, infection with HCMV is associated with lasting expansions of different NK cell subsets expressing NKG2C display memory features that can result in robust recall responses (López-Botet et al., 2014). In addition, in vaccination, expression of NKG2C might represent one potential early determinant to differentiate between responders and low responders. It can be also a driving force to promote efficacious adaptive responses at the post-vaccination stage. Indeed, vaccine responsiveness can be associated with changes in NK cell phenotype and functionality (Riese et al., 2020). In addition, NK cells present in the lung are often enriched in CD56dim/CD16bright cells but recently, during the HCMV infection, a novel adaptive killer-cell Ig-like receptor (KIR)+/NKG2C+/NK cell subset with a CD49a+/CD56bright/CD16- tissue-resident (tr) NK cell phenotype (**Figure 2**) has been identified in human lungs (Cong and Wei, 2019; Dogra et al., 2020). Considering this immunological aspect, the development of trNK cells and the emergence of their adaptive feature have also demonstrated much broader diversity than before, and the generation of different types of long-lived adaptive NK cells has added further attractiveness and complexity to this field (Freud et al., 2017; Mazarzaei et al., 2019).

### Open Questions About Effective Functions of Memory NK Cells Against SARS-CoV-2

As mentioned above, another important consideration in expanding strategies to target NK cells is related to the findings that unique tr or tissue-specific (ts) NK cell populations can display adaptive features. Recently, Marquardt et al. have identified a new adaptive-like KIR+/NKG2C+ NK cell subset with a CD49a+/CD56bright/CD16−trNK cell phenotype in human lung and in peripheral blood of HCMV-seropositive individuals, mentioned above (Marquardt et al., 2019). The adaptive-like lung trNK cells have been thus found to be hyper-responsive towards target cells (Hervier et al., 2019). This novel subset of human lung NK cells contributes to unique defense mechanisms in the context of the respiratory infection at this site (Cong and Wei, 2019). With regard to this novel adaptive-like KIR+/NKG2C+ NK cell subset, several interesting issues remain to be solved:

- Can SARS-CoV-2 infection specifically drive the expansion of adaptive-like trNK cells?
- Is disease progression in HCMV-seropositive patients involved with SARS-CoV-2 infection compared with HCMV-seronegative ones can be different? Consistent with this concept, based on clinical appearance and blood parameters during infection, we have observed that the severity of clinical manifestation in coinfection of COVID-19 and HCMV in kidney transplant patients was less severe compared with renal transplant patients with only COVID-19 (unpublished data).
- Is it possible to have a cross‐reactivity of HCMV-specific NK cells (**Figure 1**) with a protective role against new viruses (such as SARS-Cov2), even unrelated ones? Consistent with this hypothesis, previous studies have demonstrated that during acute influenza infection, adaptive-like NK cells could contribute to host immune response by promoting IFN-γ production. These cells might be clinically effective in the defense of emerging respiratory viral infections (Goodier et al., 2016; Scharenberg et al., 2019). Thus, how to target these populations might be beneficial in generating protective immunity against pathogens that gain entry through or colonize lung tissues such as novel CoV.

### Memory NK Cells to Protect Against SARS-CoV-2

A wave of investigations revealed that human NK cells presented adaptive immune responses upon immunization or infection. Therefore, NK-mediated recall responses may further allow for development of immunization-based approaches in viral treatments (Geary and Sun, 2017; Brillantes and Beaulieu, 2020). NKG2C+CD57+NKG2A-adaptive NK cells have shown effector activity in the settings of hantavirus pulmonary syndrome (HPS), chikungunya virus (CHIKV), and type I human immunodeficiency virus (HIV-1). Notably, the emergence and expansion of memory NK cells have been identified during HIV, HPS, and CHIKV infections in HCMV-seropositive patients. Indeed, evidence suggests that HCMV is responsible for the expansion of NKG2C+NK cells upon additional viral infections (Brunetta et al., 2010; Petitdemange et al., 2011; Béziat et al., 2012).

In light of the clinical implications of HCMV-induced adaptive NK cell expansions, scholars have recently reported that the expansion of HCMV-adaptive NK cells in hematopoietic stem cell transplantation may be critical to decrease the risk of relapse and enhancing graft-*versus*-leukemia (GVL) reaction in patients (Cichocki et al., 2014).

During this public health crisis, recent findings have correspondingly indicated a variation of COVID-19 susceptibility in patients, highlighting an urgent need for comprehensive risk evaluation based on memory immune responses and cross-reactivity patterns (Hamiel et al., 2020). This concept is consistent with a study that has proposed the hypothesis that the resultant immune response against prior influenza infection would, at least in part, foster immunity against SARS-CoV-2 due to this cross reactivity between Flu and SARS-CoV-2, suggesting the Flu-induced immune response could be useful in reducing the severity of COVID-19 (Salem and El-Hennawy, 2020; Salman and Salem, 2020).

In this line, HCMV infection is a common global disease with higher prevalence rates in developing countries including Iran (Yaghobi et al., 2005; Saadi et al., 2013; Marin et al., 2016). Furthermore, HCMV seropositivity has been associated with higher frequencies of NKG2C+ NK cells in peripheral blood as compared with frequencies in HCMV seronegative individuals (Davis et al., 2015). Indeed, NKG2C+ NK cells display improved ADCC functionality and IFN-γ production following exposure to antibody-coated HCMV infection cells (Hwang et al., 2012). Significant factors can accordingly contribute to modifying viral disease outcomes. Co-infection with another infectious agent including HCMV can be thus considered in terms of the influence of cross-reactivity in immune responses (Wedemeyer et al., 2001; DaPalma et al., 2010). Accordingly, what remains to be determined is whether HCMV adaptive NK cells can play a protective role against non-HCMV viral infections or not (Mirahmadizadeh et al., 2019) like this newly emerging virus (Moss, 2020). Comprehensive studies are necessary to test this hypothesis to sort out the relative influence that HCMV adaptive NK cells exert on SARS-CoV-2 pathogenicity in patients with detectable levels of SARS-CoV-2 genomic RNA (**Figure 1**). A growing body of literature also suggests a footprint of adaptive-like NK cells in other infectious agents. Moreover, memory or memory-like NK cells may expand in response to various viral and bacterial infections and some immunization strategies may arise in this way. Of note, in some experimental models, memory NK responses are clearly pathogen-specific (Brillantes and Beaulieu, 2020). For instance, NKG2C+ NK cells have expanded during the HCMV infection that result from the interaction between HCMV-derived peptide, UL40, in the context of HLA-E, and the activating receptor CD94-NKG2C (Hammer et al., 2018a). In others, NK cells may need to be reprogrammed due to inflammatory signals during infection or immunization protocol. Using HIV-envelope (Env)-vaccinated humanized bone marrow-liver-thymus (BLT) mice, Nikzad et al. had found that human NK cells could mediate robust vaccination-dependent recall responses, indicating antigen specificity and longevity (Nikzad et al., 2019). Isolating HIV-Env-primed hepatic NK cells from BLT mice had further indicated reduced expression of CD16, CD57, KIR2DL1, and KIR2DL5. It had also revealed an enhanced expression of adhesion molecules e.g. CD2, CD62L, NKG2A, and CXCR6, concluding that hepatic NK cells had been induced through DC-based HIV-Env vaccination (**Figure 2**) and had similarly altered the expression levels of different surface markers of NK cells (Paust et al., 2010; Ackerman et al., 2012; Jost and Altfeld, 2013). In addition, a study had shown that decades after initial varicella-zoster virus (VZV) exposure to varicella-zoster virus (VZV) skin test antigen in individuals experiencing VZV, adaptive NK cells had been isolated from blister fluid associated with tissue-residency including CD56hi, CD16low, and more frequently expressed CXCR6, NKG2D, CD69, and CD62L. Thus, human NK cells exhibit adaptive immune responses upon immunization or infection (Nikzad et al., 2019; Peppa, 2019).

To date, in cancer patients boosting *in vivo* NK cell-mediated antitumor activity by exposure to pro-inflammatory cytokines such as IL2, IL15, and IL18 have been identified to imprint long-lived memory-like features and to enhance NK cell survival and proliferation. In addition, IL-12 as a new prospect improves cytokine production and cytotoxicity by NK cells (Vacca et al., 2013; Uppendahl et al., 2019). Use of adaptive NK cell capabilities in cellular immunotherapies particularly in viral infection also requires clinical efforts for a better understanding of their features. It will be interesting to see in future studies whether there is a memory NK response against SARS-CoV-2 antigen-vaccinated in lung tissue samples and peripheral blood or not.

### Allogeneic NK Cells and Cellular Therapy in COVID-19

During COVID-19 emergency, efforts to confront infections have been primarily focused on cellular therapy approaches through allogeneic NK cells. Up to now, Cellularity, as a clinical-stage cell therapeutics company (NJ, USA; www.celularity.com) has produced an allogeneic and off-the-shelf cryopreserved NK cell, known as CYNK-001, derived from placental stem cells for treating leukemia and multiple myeloma (Ilic and Liovic, 2020). Promising therapeutic strategies have been also developed for patients with moderate-to-severe COVID-19. Indeed, inducing a robust immune response by allogeneic off-the-shelf NK cells may control the infection. Notably, the biology of NK cells has indicated a possibility that immunotherapy can be used as an off-the-shelf treatment for future pandemic infections and re-emerging viruses (Li H. et al., 2020). Researchers are also testing CYNK-001 because NK cells that may be able to boost immunity in COVID-19 patients as the first cell therapy are awaiting the Food and Drug Administration (FDA) approval for trials in this disease (**Figure 2**). However, it is conceivable that destroying infected respiratory cells by NK cells may drive side effects such as hyper-inflammation, as the first cell therapy awaiting FDA approval for testing CYNK-001 in COVID-19 patients. It is hoping that NK cell therapy might help patients suffering from this pandemic. Despite the fact that hyper-inflammation has not been observed while treating cancer patients with CYNK-001 (Ilic and Liovic, 2020), the mechanisms to induce the virally-driven hyper-inflammation may be different. Thus, it should be considered as a well-organized clinical trial to tune antiviral effects by escalating doses slowly while monitoring for toxicity evidence and side effects. A challenge in this regard is discovering whether COVID-19 has the ability to evade NK cells or not. However, in the global battle against COVID-19, every combat can be a valuable clue for the next step. Even though NK cells may fail to fight against COVID-19 efficiently, that lesson can be valuable to investigators.

## Perspective

Future work will need to be directed to understanding how long-lived antigen-specific NK memory responses can be targeted towards improved human health *via* the development of novel clinical diagnostic approaches, therapeutic agents, or immunotherapies. Cellular immunotherapy by cell engineering is also a relatively novel approach. A significant evolution of cell therapy can be thus represented by NK cells engineered with CAR-targeting antigens (CAR-NK) (Souza-Fonseca-Guimaraes et al., 2019). CAR-NK cells may also characterize an off-the-shelf tool based on viral immunogenic and conserved domains and even applied singly or combined with other therapeutic approaches for viruses (Mazarzaei et al., 2019). Novel strategies can be now used to manipulate NK cell function, targeting their major inhibitory checkpoints (Bi and Tian, 2019) such as KIR (Pende et al., 2009), NKG2A (André et al., 2018), and programmed cell death protein 1 (PD-1) (Lipson et al., 2013). Currently, using monalizumab (namely, NKG2A monoclonal antibody), in rheumatoid arthritis (RA) and several neoplastic disorders might be an efficient target as a new anti-SARS-CoV-2 strategy that potentially reverses the inhibition of the innate immune system induced by the virus and leads to improved NK cells cytotoxicity at the early stage of the disease. In addition, CC motif chemokine receptor 7 (CCR7) acquisition by trogocytosis to optimize NK cell traffic to lymph nodes may improve the successful outcome of alloreactive NK cells (Pesce et al., 2016). Recently, NKG2D-ACE2 CAR-NK cell therapy is being recruited for patients infected with SARS‐CoV‐2 (NCT04324996). It should be noted that NKG2D-ACE2 CAR-NK cells produce IL-15 to ensure NK cell long-term survival and release GM-CSF-neutralizing scFv to suppress cytokine storm. These cells also express ACE2 receptor to compete with SARS-CoV2 for binding sites on the susceptible cells, including alveolar epithelial cells (Bonam et al., 2020). In this line, introducing NKG2C-ACE2 into chimeric antigen receptors in NK cells to enhance effector functionality might be a potential approach in future viral immunotherapy for emerging and re-emerging viruses. In addition, novel technologies such as CRISPR/Cas9 now provide a new approach to gene editing (e.g. insertion of activating receptors or deletion of inhibitory checkpoints) (Huang et al., 2020). A recent report has further utilized induced pluripotent stem cell (iPSC) as an efficient off-the-shelf source of expanding NK cells to optimize NK-CAR engineering (Li et al., 2018).

In conclusion, a promising approach to better management of respiratory diseases, as one of the leading causes of death worldwide, may be based on remodeling NK cells phenotypic and their functional features. It is hoped that the hypotheses addressed in this review will be employed as a stimulus for further research to help combat COVID-19 as deadly contagious disease of increasing incidence around the world. The clarification of these ideas about utilizing and modifying NK cells will further allow future dissection of potential strategies for treatment and vaccination.

## Author Contributions

SS and RY conceived the idea for the manuscript. SS drafted the manuscript. RY edited and added comments into the manuscript.

## Funding

This study was supported by Shiraz University of Medical Science, Shiraz, Iran.

## Conflict of Interest

The authors declare that the research was conducted in the absence of any commercial or financial relationships that could be construed as a potential conflict of interest.
